# Strengthening and Weakening Effects of Particles on Strength and Ductility of SiC Particle Reinforced Al-Cu-Mg Alloys Matrix Composites

**DOI:** 10.3390/ma14051219

**Published:** 2021-03-05

**Authors:** Zhiyu Yang, Jianzhong Fan, Yanqiang Liu, Junhui Nie, Ziyue Yang, Yonglin Kang

**Affiliations:** 1 National Engineering & Technology Research Center for Non-Ferrous Metals Composites, GRINM Group Corporation Limited, Beijing 101407, China; yangzhiyu_grinm@163.com; 2 School of Materials Science and Engineering, University of Science and Technology Beijing, Beijing 10083, China; kangylin@ustb.edu.cn; 3 GRINM Metal Composites Technology Co. Ltd., Beijing 101407, China; liuyanqiang@grinm.com (Y.L.); niejunkey@163.com (J.N.); yangziyue@grinm.com (Z.Y.); 4 General Research Institute for Nonferrous Metals, Beijing 100088, China

**Keywords:** metal matrix composites (MMCs), strengthening mechanism, failure mechanism, material design

## Abstract

The strengthening and weakening effects of SiC particles on composite strength and ductility were studied. Al-Cu-Mg alloys matrices with three different mechanical properties were used. Their yield strength, ultimate strength, and elongation range from 90 to 379 MPa, 131 to 561 MPa, and 18% to 31%, respectively. SiC particles with sizes of 4, 8, 12, 15, 20, and 30 μm were used to reinforce these three matrices, separately, and the composites of eighteen combinations of the particle sizes and matrix strengths were manufactured. Yield strength, ultimate strength, elongation, and fracture morphology of these composites were characterized. Based on the analysis, the strengthening to weakening behavior on strength and ductility were comprehensively discussed. The critical particle size having the best ductility was obtained. The strengthening limit and match range of the particle and the matrix to achieve effective strengthening were defined as a function of the particle size and matrix strength. This work offers an important reference for optimization of mechanical properties of the particle-reinforced metal matrix composites.

## 1. Introduction

Particle-reinforced metal matrix composites (PRMMCs) are attractive alternatives for applications that need a combination of high specific strength, and specific stiffness and ductility, such as new energy vehicles, aircrafts, and devices in aerospace [1,2,3]. However, the reinforcement can also reduce ductility, which limits the application in structural components. To meet the needs for advanced composites, it is important to achieve a relatively high ductility when pursuing a higher-strength composite. To achieve higher performance, many new reinforcements and matrices are matched to obtain ideal combinations of the composite [4,5,6]. The existence of the particle reduces the ductility, which limits the application of the PRMMCs in the engineering field. Furthermore, when the particle is matched with an improper matrix, the effect of the particle may change from strengthening to weakening [7,8,9]. With an improper match of the particle and the matrix, the strength of the composite may be lower that of its matrix. Besides, when the particle size increases, the elongation of the composite can either increase [7,8,10,11,12,13] or decrease [8,10,11,14] in different strength matrices. This phenomenon also makes it difficult to obtain an ideal combination of high strength and relatively high ductility without a large number of experiments.

Besides developing new composites, many efforts have also been made to improve existing PRMMCs by optimizing the match of the matrix and the particle. Many particle factors can influence the composite properties. Generally, the composite strength increases with decreasing particle size [15,16,17], or increasing particle volume fraction [18,19,20], aspect ratio [19,21], and particle strength [22,23]. The distribution of the particle can also influence the mechanical properties of the composite. With a homogeneous or directional distribution, the strength and ductility of the composite can be improved [24,25,26]. However, the prediction of strengthening to weakening behavior of the particle on strength has not been performed in existing studies. The dominant factor of these two opposite behaviors of the particle, strengthening and weakening, needs to be revealed. A comprehensive study of the effects of the particle on the strength and the ductility is still needed.

The aim of this study was to provide a better understanding of the effects of reinforcement on mechanical properties and a new perspective to obtain a better combination of strength and the ductility of the PRMMCs based on SiCp/Al-Cu-Mg alloys composites. The eighteen composites combined with SiC particles of six different sizes (4, 8, 12, 15, 20, and 30 μm) and Al-Cu-Mg alloys matrices of three different strengths (yield strength of 90, 292, and 378 MPa) were manufactured. Yield strength (YS), ultimate strength (UTS), elongation (EL), and fracture morphology of these composites were measured. Based on the analyses of these data, the strengthening to weakening mechanisms and their effect on strength and ductility were discussed. The size of the particle to achieve maximum ductility for these composites was obtained. The match range of the particle size and the matrix strength to determine the effective strengthening of the composite were defined using the polynomial fitting, which could help in developing a new PRMMC in the materials selection stage.

## 2. Materials and Methods

Average α-SiC particles sizes of 4.1, 7.7, 11.3, 14.8, 19.6, and 29.9 μm were used to reinforce the matrices, which cover the effective strengthening particle size and are sufficiently large to causing a weakening effect. The Al-Cu-Mg alloy, which was combined with a wide range of strength and elongation, was used as matrices. According to the Al-Cu-Mg ternary phase diagram, when the mass ratio of Cu to Mg is 2.61:1, the alloy phase is almost Al_2_CuMg. This can put the matrix in a more simple state where the fewest types of precipitate exist. By altering the amount of Cu and Mg and keeping the Cu:Mg mass ratio of 2.61:1, matrices with different mechanical properties were obtained. These three matrices are named L, M and H based on the level of their strength, which represent low, medium and high level of strength of the matrix. The compositions and mechanical properties of three matrices are shown in Table 1. The 15 vol% of SiC particles was mixed with highly pure aluminum, magnesium, and copper powders in a ball miller with a ball-to-powder weight ratio of 2:1 for 24 h. The ball used for the milling process was made of steel and had two different sizes of 5 and 2 mm (weight ratio 1:1). The barrel of the miller had a depth of 300 mm, diameter of 200 mm, and angle of 45° to the rotating axis. The powder and balls were sealed in a barrel with air condition and rotated with a speed lower than 60 rpm. The mixed powder was degassed and processed by hot isostatic pressing at 520 °C. Then, the composite billet was hot-extruded into rods with a diameter of 30 mm at 520 °C. After that, they were processed with heat treating at 520 °C for 2 h, water quenched and aged at room temperature for 120 h before being shaped and tested. The composites were named by their combinations as matrix number-approximate particle size; for example, the composite H-30 represents matrix H reinforced by 29.9 μm particles.

The composite rods were shaped into a tensile specimen with a gauge length of 30 mm and subjected to uniaxial loading at a strain rate of 0.5 mm/min on an AMSLER-100-20 universal testing machine (ZwickRoell, Kennesaw, GA, USA). A clip-on extensometer was used to measure the strain. The yield strength was defined as 0.2% offset yield strength. Four specimens of each composite were tested. The mechanical properties used in this work were the average values of 4 tests’ results.

The metallograph was photographed using a Zeiss Axiovert 200 MAT (Carl Zeiss, Oberkochen, Germany). The fracture images were photographed using a JEOL JSM-7900F scanning electron microscope (SEM) (JEOL Ltd., Tokyo, Japan). The EDS image was photographed by an EDS module (Octane Elect Super, EDAX, LLC, Mahwah, NJ, USA) on SEM. The software Image Pro-Plus (Media Cybernetics, Inc., Rockville, ML, USA) was used to count the dimple size from the SEM images. Five images at a magnification of 5000× (23 μm × 13 μm) for each composite with matrices M and H and magnification of 2000× (61 μm × 34 μm) for a composite with the L matrix were used to count the dimple size, and the results in this paper are the average values of 5 images’ results. At least 100 dimples for composites with matrices M and H and 70 dimples for composites with matrix L were measured.

## 3. Results

### 3.1. Mechanical Properties

**Figure 1:**
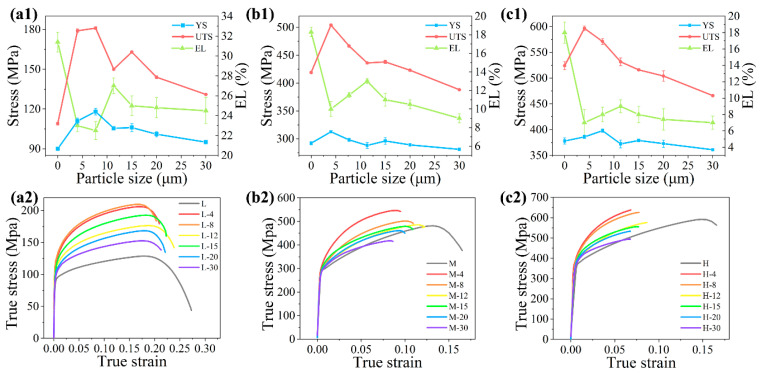
The (**1**) mechanical properties (yield strength (YS), ultimate strength (UTS), and elongation (EL)) and (**2**) true stress–true strain curve of the composites with (**a**) matrix L, (**b**) matrix M, and (**c**) matrix H reinforced by particle sizes of 4.1, 7.7, 11.3, 14.8, 19.6, and 29.9 μm, separately. The 0 μm particle size represents the unreinforced matrix.

The YS, UTS, EL, and true stress–true strain curves obtained from the uniaxial tensile testing are shown in Figure 1. The composite yield strength and the ultimate strength (YSc and UTSc, respectively) decreased with the increasing particle size. When the particle size in matrices M and H exceeded 15 and 8 μm, the YSc values were lower than that of YSm (Figure 1b,c). The same phenomenon was also be found in the UTSc with matrix M and H (Figure 1b,c). The same strengthening to weakening transition on composite strength can be found when the matrix strength increases with a given particle size. This means the strengthening effect of one size particle has its limit on composite strength. When the strength of the matrix exceeds the strengthening limit of the particle, the effect of the particle will change from strengthening to weakening.

The variation trend in the EL was found to be more complex compared to that of the strength. When the particle size was smaller than 12 μm, the values of EL increased with increasing particle size. However, the EL values decreased with the increasing particle size when particle size exceeded 12 μm. This indicated that different factors dominate the variation in EL in different size range of the particle.

### 3.2. Microstructure

**Figure 2:**
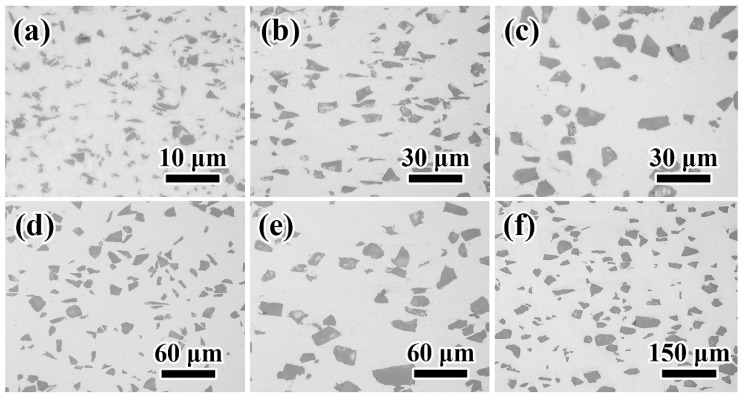
The typical metallograph of (**a**) H-4, (**b**) H-8, (**c**) H-12, (**d**) H-15, (**e**) H-20, and (**f**) H-30.

Typical metallographs of composites with the H matrix are shown in Figure 2. Figure 1 shows that all sizes of particles were well-dispersed. The large particles were more uniformly distributed than the particle with small size. The agglomeration of the particle was found to be less. The long axis of the particle was parallel to the extrusion direction (horizontal direction) due to the extrusion.

**Figure 3:**
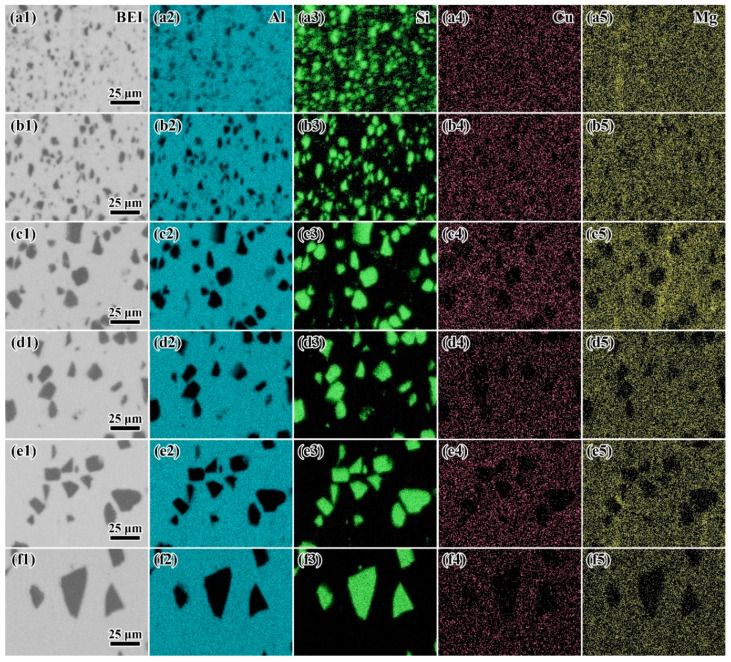
The typical EDS mapping of (**a**) H-4, (**b**) H-8, (**c**) H-12, (**d**) H-15, (**e**) H-20, and (**f**) H-30. The results of (**1**) backscattered electron image, EDS mapping of (**2**) Al, (**3**) Si, (**4**) Cu, and (**5**) Mg are shown in every group.

The distribution of elements is shown as EDS mapping in Figure 3. As shown in the figure, Si and SiC particles had the same distribution. The elements Cu and Mg followed the distribution of the element Al. This indicated that the major alloying elements Cu and Mg had a uniform distribution. No obvious agglomeration was found.

### 3.3. Fracture Morphology

**Figure 4:**
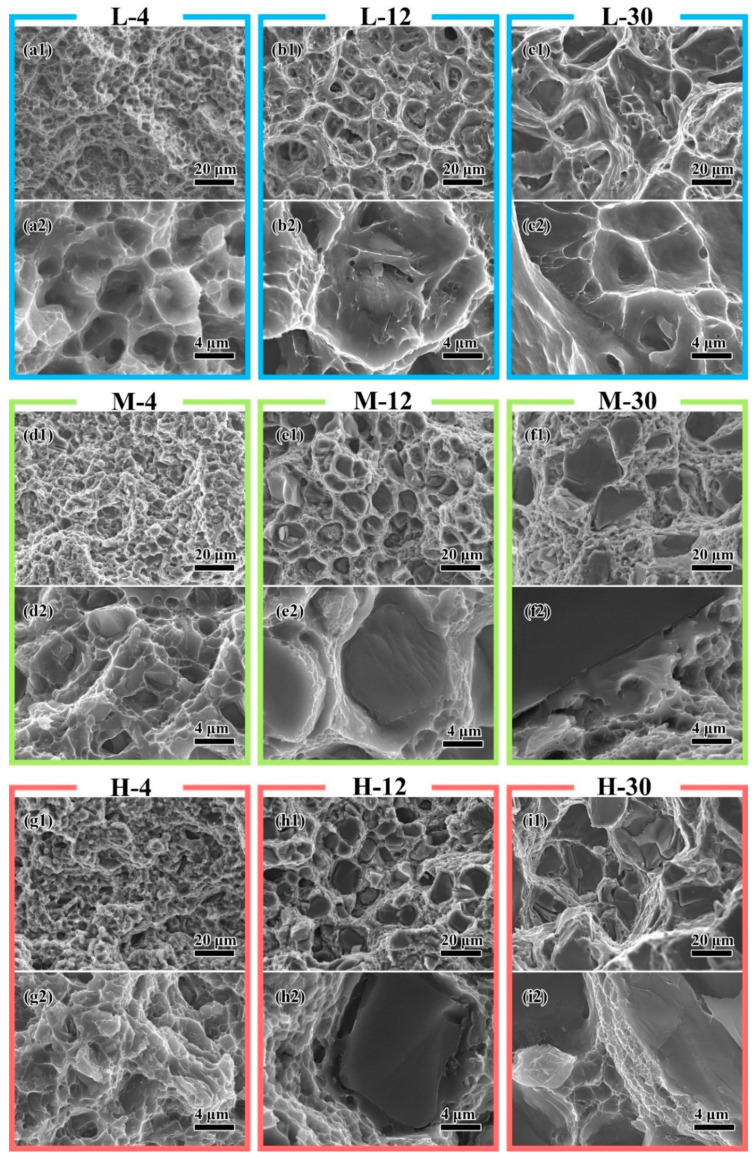
The typical fracture morphologies of (**a**) L-4, (**b**) L-12, (**c**) L-30, (**d**) M-4, (**e**) M-12, (**f**) M-30, (**g**) H-4, (**h**) H-12, and (**i**) H-30. The two images in each group are the images with different magnifications of (**1**) 1000× and (**2**) 5000×.

The fracture surfaces of all composites were observed and photographed by SEM. To simplify the presentation of the results, the fracture morphologies of nine typical composites are shown in Figure 4. With increasing particle size and matrix strength, the particle cracking on the fracture surface increased.

In the composites with a low-strength matrix, the fracture surface was composed of large size dimples (Figure 4a–c). The dimple size of the matrix increased when particle size increased from 4 μm (Figure 4(a2)) to 12 μm (Figure 4(b2)); however, it decreased when particle size further increased to 30 μm (Figure 4(c2)). In L-4, almost no broken particles were found on the fracture surface (Figure 4(a1)). The large dimples and tear ridges showed the void coalescence failure mechanism (Figure 4(a2)). When the particle size increased to 12 μm in L-12, cracked particles were still hardly found (Figure 4(b1)). However, at the bottom of large dimples, particles with a layer of matrix on their surfaces were found (Figure 4(b2)). This indicated that void coalescence at the particle surface generates large cavities. When these cavities grow and connect, catastrophic failure happens. With the continually increasing particle size, broken particles were found at the bottom of large dimples in L-30 (Figure 4(c1)). The unbroken particles were also found in some small dimples (Figure 4(c2)). This indicated that a large level of deformation happens around the cracks of the particles, connects the cavities around particles, and generates the main crack. Matrix-induced damage is the dominant factor in low-strength matrix composites.

When high-strength matrices were used, the dimple size significantly decreased, and the number of broken particles increased. On the fracture surface of M-4 and H-4, a few cracked particles were found (Figure 4(d1,g1)). The transition from the fracture surface of the particle to matrix was flat and smooth (Figure 4(d2,g2)), which means a fast crack propagation occurred. Particle cracks soon induced the catastrophic failure at a high level of stress. When particle size increased, a smooth surface was found around the particle (Figure 4(e2,h2)). These microstructures indicated that after a particle is broken, the crack of the particle grows slowly with the composite deformation. In the composite with large particles and a high-strength matrix, small and shallow dimples between the broken particle were found. This indicated that too many particles cracked and connected to form the main crack before enough deformations of the matrix occurred to reduce the concentrate strength. Particle-induced damage is the dominant factor in high-strength matrix composites.

**Figure 5:**
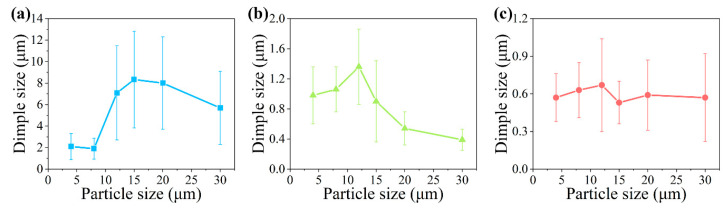
Average dimple sizes on the fracture surfaces of the composites with (**a**) matrix L, (**b**) matrix M, and (**c**) matrix H. The error bars show the variation range in the dimple size in one composite.

The dimple sizes on the fracture images were counted and the results are shown in Figure 5. Dimple size increased with the increasing particle size before the 12 μm particle size. When particle size was larger than 12 μm, the dimple size decreased. The dimple size also decreased significantly with increasing matrix strength. These changing trends are similar to those of elongation shown in Figure 1.

## 4. Discussion

### 4.1. Effects of the Particle on Strength

The effects of the particle on YSc and UTS are different. Many effects caused by the particle strengthen the YSc. The loading-bearing effect [27,28], the Orowan strengthening [29], the strengthening caused by the thermal expansion mismatch [30], and plastic deformation mismatch [31] are the main contributions to YSc. According to the analytical expressions of these contributions in the related references, these contributions to YSc increase when the particle size decreases. When a high-yield-strength matrix is used, the yield strength of the composite increases [32]. With an improper high-strength matrix is used, the stress on the particle may exceed the particle strength, which would lead to particle cracking. The particle cracking reduces the actual loading area, which makes the engineering stress calculated from the cross-section of the original specimen lower than the actual stress. The decline in the number of particles will also reduce the strengthening effects of loading transfer and dislocation strengthening, which reduce the obstacles of dislocation and the yield strength of the composite. When particle cracking occurs before the yielding of the composite in one combination of a high-strength matrix and relatively large particle, the yield strength of the composite will decrease and become even lower than that of its matrix [33], which occurred in M-30, H-20, and H-30 in this research.

The ultimate strength is mainly strengthened by the strain-hardening effect and is sensitive to the defects in the composite. The existence of the particle increases the strain-hardening effect [34,35]. Conversely, the particle will also continually crack during deformation of the composite [10,32,36], which reduces the contributions to the composite strength and the effective loading area of the specimen. The higher the matrix strength or the larger the particle size, the more particle cracking occurs [32,33,36]. The particle-cracking-induced damage reduces the flow stability of the composite, which usually causes early fracture [11,37]. It can also be seen from the true stress–true strain curves in Figure 1. Three matrices all had the necking part in the final stage of the deformation. With increasing matrix, the necking parts shortened, as shown in Figure 1b2, and nearly disappear in Figure 1c2. The early fracture of the composite becomes obvious when the strength of the matrix increased, which caused the UTSc of M-20, M-30, H-15, H-20, and H-30 to be lower than that of their matrices. The effect of the particle on the strength is decided not only by the performance of the particle, but also by the matrix. Using a proper match of the particle and the matrix can lower the particle-induced damage and obtain a good strengthening effect of the particle.

**Figure 6:**
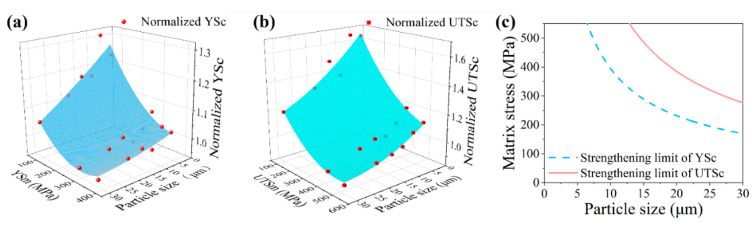
The relationship of (**a**) normalized composite yield strength (normalized YSc) and (**b**) normalized composite ultimate strength (normalized UTSc) with the particle size and the matrix yield strength and the ultimate strength (YSm and UTSm), respectively. The normalized strength is normalized to the strength of its matrix. (**c**) The strengthening limits of the composite strength obtained by numerical fitting. The matrix strength on the Y axis represents the YSm or UTSm when the strengthening limit line of YSc or UTSc is used.

The normalized strength (normalized to the strength of its matrix) can be considered the representative of the particle-strengthening ability. The normalized YSc and UTSc are shown in Figure 6a,b, respectively. It can be seen that the normalized strength increases with decreasing matrix strength and particle size. The smaller particle has a higher strengthening ability. Notably, the strengthening ability has its limit. When the matrix strength increases to obtain a normalized strength below one, this matrix cannot be reinforced by this particle. The effect of the particle on matrix changes from strengthening to weakening.

A polynomial is used to fit the relationship of normalized strength with the matrix strength and the particle size:

Ns = z − aD − bσ + cD^2^ − dσ^2^ + eDσ (1)

where Ns is the normalized strength, D is the particle size, and σ is the matrix strength. The fitting results of normalized YSc and UTSc are shown in Table 2, and Figure 6a,b, respectively. Based on Equation (1), the strengthening limits of YSc and UTSc can be calculated by determining the Ns value to one, and the results are shown in Figure 6c. As the YS is usually considered in materials design, the matrix and the particle used should have a normalized YSc value over one to obtain an effective strengthening result.

### 4.2. Effects of the Particle on Ductility

The elongation of the composite increased with the increasing particle size when the particle size was smaller than 12 μm. After the particle size increased up to 12 μm, the elongation of the composite began to decrease with increasing particle size. The particle size has a critical size in a composite to obtain the maximum elongation value. When a composite with a small particle deforms, the strong resistance of the particle to the dislocation movement limits the matrix deformation into small regions where the tiny cavities are generated [33]. In the high-level stress state, these tiny cavities and cracks of the particle connect and the main crack is generated. The fracture surface composed of many tiny dimples is formed as shown in Figure 4d,g. When particle size increases, this resistance decreases, and the deformation ability increases. The voids in a wider range can aggregate, and the dimple size increases, as shown in Figure 5. However, the increase in the particle size also reduces the particle strength, which increases the particle cracking [38]. With increasing particle cracks, the dominant failure mechanism factor changes from matrix-induced damage to particle-induced damage [33]. The increasing particle cracking will decrease the deformation stability and lead to early fracture of the composite [11]. The earlier the fracture occurs, the less the growth of the dimple, which leads to the smaller size of the dimple, as shown in Figure 5. The interaction of these two factors determines the rise–fall changing character of the composite elongation. When a composite is designed to have good ductility, the particle should have a size close to the critical size. In this work, the critical size was approximately 12 μm.

## 5. Conclusions

In this study, the effects of the particle on the strength and ductility of SiC particle- reinforced Al-Cu-Mg alloys matrix composites were investigated. Yield strength, ultimate strength, elongation, and fracture morphology of composites with eighteen combinations were characterized. The dimple sizes on the fracture surface were also counted from the fracture surface images. Based on the analysis of these results, the following conclusions can be drawn:

(1) The strengthening ability of the particle decreases with increasing particle size as well as the matrix strength, and has a strengthening limit. When the particle size exceeds the limit, the effect of the particle changes from strengthening to weakening.
(2) The strengthening ability and its limit were defined as a function of the particle size and the matrix strength using polynomial fitting.
(3) The elongation of the composite increases with the increasing particle size when the particle size is smaller than the critical size. The main factor in this stage is the resistance of the particle to the matrix deformation. When particle size is larger than the critical size, the composite elongation decreases with the increasing particle size due to the early fracture caused by particle cracking.

## Figures and Tables

**Table 1:** Chemical compositions and mechanical properties of the matrices. YS, yield strength; UTS, ultimate strength; EL, elongation.

Matrix	Chemical Compositions (wt %)			Mechanical Properties		
	Cu	Mg	Al	YS (MPa)	UTS (MPa)	EL (%)
L	-	-	100.0	90	109	31.4
M	2.6	1.0	Bal	292	419	18.3
H	4.2	1.6	Bal	378	524	18.0

**Table 2:** Fitting results of the relation of normalized strength with the matrix strength and the particle size.

Grops	z	a	b	c	d	e	R^2^
Normalized YSc	1.48	−1.17 × 10^−2^	−2.09 × 10^−3^	4.28 × 10^−5^	2.40 × 10^−6^	2.19 × 10^−5^	0.94
Normalized UTSc	2.03	−2.90 × 10^−2^	−2.68 × 10^−3^	2.69 × 10^−4^	2.06 × 10^−6^	2.18 × 10^−5^	0.95

## Data Availability

Data are contained within the article.
